# Development of a Biomechanical Device for Parameter Quantification Associated with the Sit-to-Stand Movement

**DOI:** 10.3390/s23041837

**Published:** 2023-02-07

**Authors:** Natacha Oliveira, Filipe Carvalho, Jorge Laíns, Deolinda Rasteiro, Luis Roseiro

**Affiliations:** 1 Polytechnic of Coimbra, ISEC, Rua Pedro Nunes, Quinta da Nora, 3030-199 Coimbra, Portugal; 2 Centro de Medicina de Reabilitação da Região Centro, Hospital Rovisco Pais, 3064-908 Tocha, Portugal; 3 Applied Biomechanics Laboratory, i2A-IPC, Rua Pedro Nunes, Quinta da Nora, 3030-199 Coimbra, Portugal; 4 Faculty of Medicine, University of Coimbra, Rua Larga, 3004-504 Coimbra, Portugal; 5 Center for Mechanical Engineering, Materials and Processes (CEMMPRE), University of Coimbra, Pinhal de Marrocos, 3030-788 Coimbra, Portugal

**Keywords:** motor rehabilitation, sit-to-stand movement, biomechanics

## Abstract

The “sit-to-stand” (STS) movement is essential during activities of daily living (ADL). In individuals with physical-motor diseases, its execution and repetition increases activity levels, which is crucial for a good motor rehabilitation process and daily training. Interestingly, there are no sit-to-stand devices that allow a quantitative assessment of the key variables that happen during STS, and there is a need to come up with a new device. This work presents a developed biomechanical support device that measures the force of the upper limbs during the STS movement, aiming to motivate and encourage people undergoing physical therapy in the lower limbs. The device uses two instrumented beams and allows real-time visualization of both arms’ applied force and it records the data for post-processing. The device was tested with a well-defined protocol on a group of 34 healthy young volunteers and an elderly group of 16 volunteers from a continuing care unit. The system showed robust strength and stiffness, good usability, and a user interface that acquired and recorded data effectively, allowing one to observe force-time during the execution of the movement through the application interface developed and in recording data for post-processing. Asymmetries in the applied forces in the STS movement between the upper limbs were identified, particularly in volunteers of the continuing care unit. From the application and the registered data, it can be observed that volunteers with motor problems in the lower limbs performed more strength in their arms to compensate. As expected, the maximum average strength of the healthy volunteers (both arms: force = 105 Newton) was higher than that of the volunteers from the continuing care unit (right arm: force = 54 Newton; left arm: force = 56 Newton). Among others, moderate correlations were observed between weight-applied and height-applied forces and there was a moderately high correlation between the Sequential Clinical Assessment of Respiratory Function (SCAR-F score) and time to perform the movement. Based on the obtained results, the developed device can be a helpful tool for monitoring the evaluation of a patient with limitations in the upper and lower limbs. In addition, the developed system allows for easy evolution, such as including a barometric platform and implementing serious games that can stimulate the execution of the STS movement.

## 1. Introduction

Old studies show the complexity of animal movements. Control and coordination in all body system components are needed to carry out an activity [1]. In the case of humans, the study of movement is essential to understand various contexts associated with everyday life. Over the past few centuries, several vital advances have substantially impacted the perception of human movement, which has significantly evolved in recent years through technological development and the availability of analysis tools. During the 1950s, the study of human activity became more critical due to the need to treat citizens and soldiers who were disabled in the Second World War, increasing this investigation [2].

Nowadays, the increase in life expectancy is one of the factors that motivates researchers to observe and interpret human movement. Over the years, several studies have presented devices and technologies that focus on human movement [3]. These studies start with the parameterization of different tasks to classify human movement and the phases that involve their execution. The need to study the body’s behavior, actions and reactions are the most important reasons for studying human movement, which allows researchers to apply this knowledge in developing devices and new techniques that improve human health. Implementing rehabilitation methodologies associated with the human body or recovery is possible. Observing locomotion tasks allows an understanding of the difference between standard and pathological functions and shows how to prevent and treat injuries. As an example, some direct applications can be identified in orthopedics, sports or physical medicine, and rehabilitation, identifying and implementing technical aspects linked to ergonomics [3,4,5].

It is possible to identify several pathologies that directly affect a patient’s mobility, such as those associated with diseases such as stroke, Parkinson’s disease and multiple sclerosis, which present problems with strength, flexibility and movement control [6]. The older population also presents mobility limitations due to loss of muscle mass. Pain in the joints and the lower limbs are typical in the elderly, decreasing their strength and range of motion and limiting the achievement of several movements, including standing up and sitting down. These limitations represent a risk for the person, as they can result in falls, which cause injuries, fractures and even death in extreme cases [1,7,8,9,10].

After coronary artery disease and cancer, stroke is the third most common cause of death. After age 55, the probability of a stroke starts to increase significantly. In the United States of America, there are about 700,000 strokes a year; in total, 40% of survivors have a minor disability or disability and 15% to 30% are severely disabled [11]. Some studies report that 23% to 50% of people in the post-stroke state suffer an episode of fall every year, which means a rate incidence higher than healthy older adults of the same age [12]. Post-stroke patients show a more unstable posture than healthy individuals of the same age due to postural sway during static and dynamic balance tasks [13]. Falls are common in the elderly population, and it has been estimated that 28% to 35% of outpatients who are at least 65 years old experience a fall each year, [14]. Biomechanically, static balance can be defined as the capacity to maintain the center of mass on a static support base with a minimal oscillation posture.

In contrast, dynamic balance is the ability to maintain the body with a stable posture while the center of the mass and the base of support are moving. The primary purpose of rehabilitation in post-stroke patients involves improving the motor performance of the patients in activities of daily living (ADL), requiring a reliable and valid evaluation that quantifies the state of the patient’s motor functions and mobility for motorizing their progress [3,4,5]. Falls are the leading cause of injuries and limitation of activities in the elderly population, and the associated adverse effects result in personal, social and economic constraints. Approximately 30% of people 65 years of age and over who live in the community suffer a fall once a year. Falls are 40% of the causes of death due to injuries and induce 20–30% of severe injuries to fractures in the elderly. They can derive from several extrinsic (related to the environment), intrinsic (corresponding to the person) and behavioral (related to the activities developed) factors [15]. Most of the falls happen when older people are performing tasks such as transfers and walking [16,17].

### 1.1. Timed Up and Go (TUG) and Sit-to-Stand (STS) Movements

Human beings carry out various activities unconsciously during their daily lives. When the motor system is in perfect condition, it has no difficulty carrying out these activities, but it is more challenging to make certain moves if that is not the case. Most tasks that are physically demanding throughout the day begin with the person sitting and intending to get up to accomplish another task. This lifting movement is called “sit-to-stand” (STS). To successfully carry out this movement, the individual requires strength and body coordination. People with pathologies often have difficulty balancing, so performing the STS movement successfully is more challenging.

The Timed Up and Go test (TUG) was developed in 1991 as a modified and timed version from the Get Up and Go test [15] and allowed professionals to estimate the risk of falling in older adults [18,19,20]. The test quantifies the time associated with a set of movements: getting up from a chair; walking 3 m; turning; and sitting in the chair [20].

The TUG is a multifaceted measure of functional mobility. It includes transfer tasks (standing up and sitting down), walking and turning and incorporating neuromuscular components such as power, balance and coordination [21]. The cutoff level for TUG is 13.5 s, with an overall correct prediction rate of risk for falling of 90% [18]. The study of Nachtegaal and Allum [22] aimed to determine how trunk sway measurements during the TUG test could distinguish fallers from non-fallers, and concluded that non-fallers are significantly quicker than fallers performing the TUG test. The TUG test has been used in patients with stroke, is a valid clinical test to evaluate functional mobility and is reliable when applied to individuals with stroke [23].

The “sit-to-stand” (STS) test is commonly used to assess the level of mobility of the individual and so estimates the ability to get up from a sitting position to reach the standing position [24]. Performing this test helps improve human movement and allows physicians to define and indicate therapy movements for each individual. This test is considered a viable method to determine the loss of balance [1].

The five times sit-to-stand test (5STS) is often used as a functional measure of strength, but other studies have shown that factors other than muscle strength influence performance. The 5STS test has been associated with postural balance disorders and cognitive function [25,26]. This test is reliable in individuals following a stroke [27,28]. Duncan et al. [26] suggested that strength and standing balance have a significant relationship with 5STS time, independent of each other. This study indicated that balance and strength contribute to 5STS time after stroke. Maeda et al. [29] concluded that STS motion is valuable for identifying walking ability in chronic stroke patients. Postural sway after STS motion is closely related to gait and balance ability in stroke patients. Recently, some studies have examined using 5STS to identify balance disorders and those at higher risk of falling, such as older people [28]. A recent study [30] demonstrated that older adults with a history of falls had more variability in their STS transition during the 5STS test compared with younger and older non-faller participants. More variability in simple daily functional tasks such as sit-to-stand can indicate motor control deficit and dynamic impairment, leading to an increased risk of falling in older people.

### 1.2. Sit-to-Stand Devices

A walker is a device developed to support and maintain balance or stability while walking. Based on a lightweight frame system, the first walkers appeared in the early 1950s. Other variants, such as the registered invention of a walking aid [31], appeared after that. The author’s idea was to support or assist individuals who have lost the ability to walk. Nowadays, several adjustable walkers can be found in the market.

Chugo et al. [32] developed a support system for older people whose principle is to help patients with the STS movement. The device incorporates a “support pad” with three degrees of freedom and an active system to support locomotion. When the patient gets up with the device’s help, they lean on the “pad”, managing to maintain their posture without fear of falling. The “pad” is instrumented with two strain gauges, allowing for quantification of the pad’s strength and the armrest’s strength. The authors used an instrumented system with a walker as a support structure to assist the STS movement. Additionally, they intended to monitor the positions of the ankle, knee, hip, shoulder, and wrist, recorded through images using visual analysis markers. The STS movement is performed with the aid of a fixed support, such as a walking aid, instrumented with strain gauges in the area connected to the handle to record the force applied by the hands during the STS movement.

### 1.3. Proposed Device for Sit-to-Stand Evaluation

Currently, there is a lack of sit-to-stand devices that allow a quantitative assessment of the key variables that happen during STS, and there is a need to come up with a new device. This work proposes a simple biomechanical device to help individuals during the STS movement. The system can be used to quantify and evaluate a set of parameters associated with the STS movement, particularly the force time applied from the upper limbs. The system also has one application to motivate individuals with pathologies, encouraging them to undergo physical therapy in the lower limbs, namely performing the STS movement.

## 2. Materials and Methods

### 2.1. Measurement System

The developed device to support the STS movement was based on two AISI 304 tubular instrumented beams with an external diameter of 35 mm and 2.5 mm of thickness. The beam was chosen to have the necessary strength, stiffness and ergonomic dimensions for the handgrip handle [33]. The two beams of the system had height and width adjustments to guarantee their use by individuals with different anthropometric characteristics, including wheelchair users.

Each beam was instrumented near the fixed part with four strain gauges, aligned with the axis, and positioned 0°/90°/180°/270°. The strain gauges allowed us to obtain the local linear deformation of the beam (in microstrain—με) that, through a calibrated procedure, was converted into the force applied in the vertical plane (gauges 90°/270°), horizontal plane (0°/180°) and the resultant.

**Figure 1:**
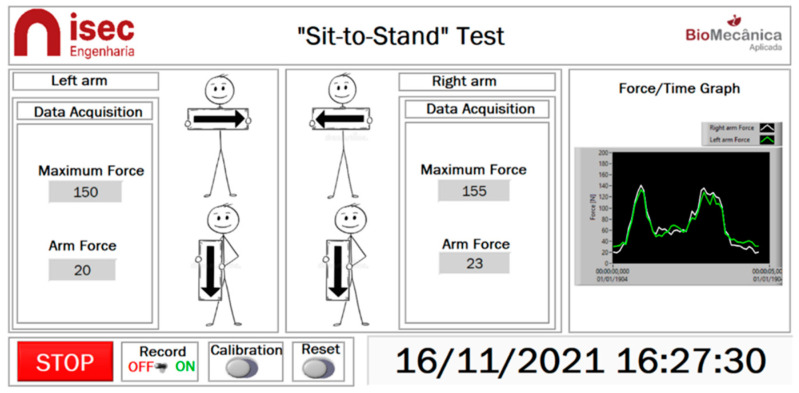
Visualization of user interface of the application.

The system incorporated a National Instruments chassis (NI-cDAQ-9174) and two data board acquisitions (NI 9219), ensuring a total of eight channels necessary to connect all the strain gauges (each one in a quarter bridge). The acquisition of the signal from the strain gauges was carried out through the LabView software. Two applications were developed in this software, one for the system’s calibration and another for the user interface to the system and data recording during the use of the biomechanical system in the STS movement. The application allowed for recording the applied force over time, the visualization of the maximum applied force by each arm, and the resultant force’s direction. The force-time can also be visualized in real-time. Figure 1 shows the interface with the user. The forces applied by each arm were recorded in an Excel file for post-processing.

**Figure 2:**
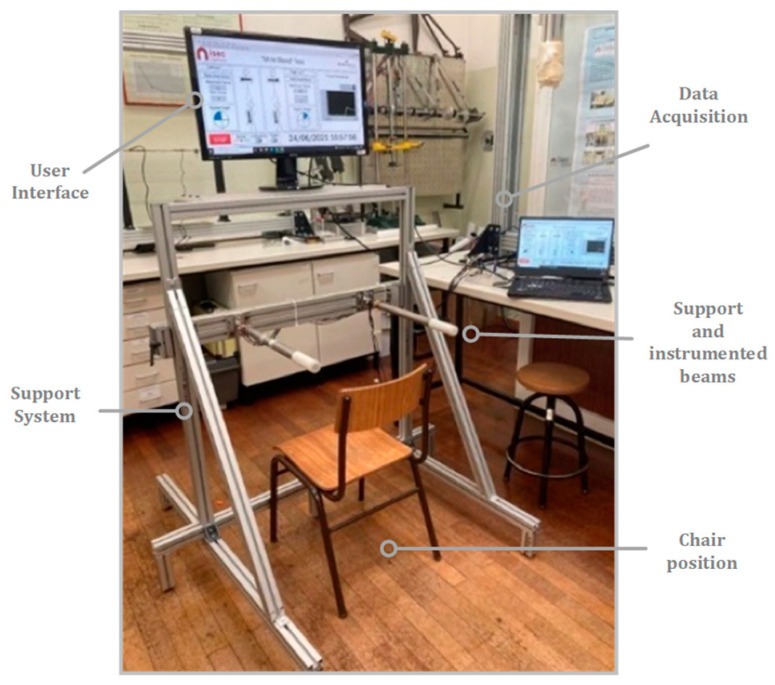
Experimental setup of the device (movable).

The anchoring elements for the measuring system can be fixed in a wall or movable and were produced with a technical Minitec Profile 45 × 45. Figure 2 shows the transportable anchorage with the measuring system positioned and fixed. The anchoring system’s upper part allows the user interface placement through a screen or laptop. The anchorage system can easily be dismantled for transport.

### 2.2. Test of Device—Volunteers

The device validation involved experimental tests associated with the STS movement. Two groups of volunteers were considered in the experimental evaluation: 34 healthy young university students (Table 1) and 16 older people from a continuing care hospital unit (Table 2).

The tests with the healthy young volunteers were intended to evaluate the device and obtain an STS pattern of the results for the device involving individuals without pathologies. For this purpose, a data acquisition protocol was defined. The authorization for the test was obtained from the ethics committee of the Polytechnic Institute of Coimbra (reference 119_CEPC2/2020).

The tests with the group from the continuing care unit were intended to evaluate the device with this type of population and compare the results from STS performed with those obtained from the healthy volunteers. Additionally, an experimental acquisition protocol was defined, and authorization for the test was obtained from the ethics committee of the Rovisco Pais Hospital (approval written on 22 July 2021).

### 2.3. Test of Device—Procedures and Measurements

The tests performed by young and healthy volunteers were carried out in two positions: one with the chair facing the front of the device and the other with the chair facing the back. A total of ten records were taken per volunteer, five in each position, with one minute of rest between records. An office chair with vertical adjustment and lateral support for the arms was considered, being as the seat could be adjusted to obtain the lower limb flexed at 90°. Additionally, the position of the beams was adjusted for each volunteer, namely their height or distance to the floor, corresponding to a slight arching of the upper limbs and its opening aligned with the distance between the hands for the adjustment position. The volunteer was then asked to use the device as support during the STS movement.

**Figure 3:**
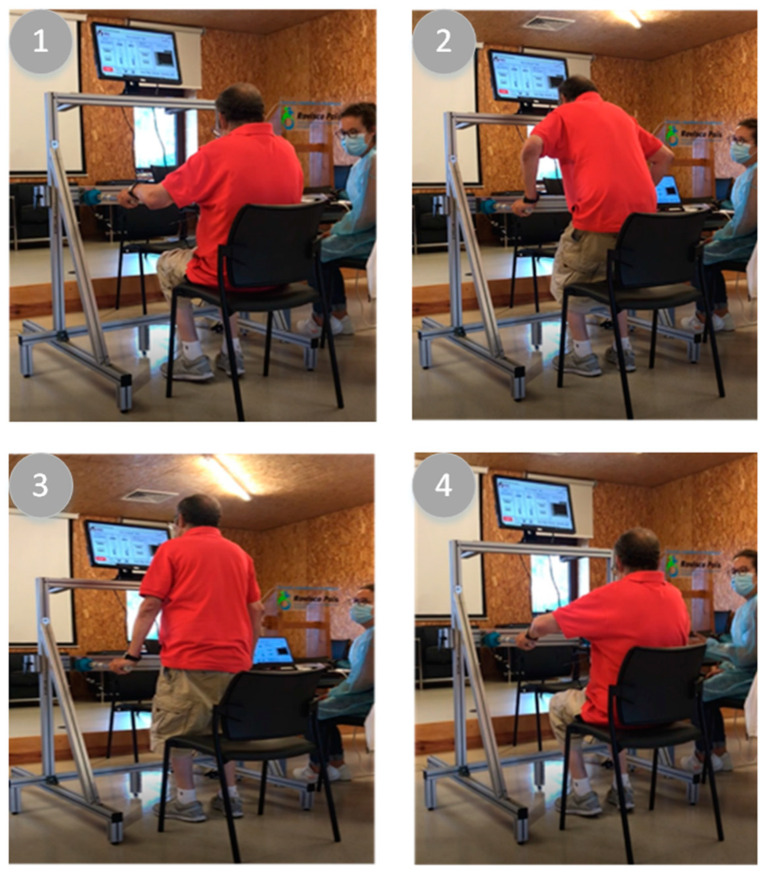
Example of experimental tests carried out by the volunteers from the continuing care unit: (**1**) Sitting; (**2**) Standing up; (**3**) Standing; (**4**) Sitting down.

Considering the test with volunteers from the continuing care hospital unit and the gait difficulties (Table 2), it was defined for the volunteers to repeat the STS movement only three times and with the chair facing forward (example in Figure 3), which was considered enough to obtain the necessary results. This test used a fixed padded chair without lateral support for the arms and with equal height for all volunteers. As for healthy volunteers, the vertical position of the beams was adjusted, corresponding to a slight arching of the upper limbs and its opening aligned with the distance between the hands. The volunteer was asked to use the device as a comfort support during the STS movement.

Two parameters were added for analysis: the time it took to perform the STS movement and the SARC-F (sarcopenia risk screening tool). SARC-F is the loss of muscle mass, being classified through scoring through a questionnaire answered by the volunteers. A score greater than four shows the probability of sarcopenia [34].

### 2.4. Data Recording and Analysis

For each test performed, the applied force by each arm was recorded during the time of execution of the STS movement. This record was time-synchronized and saved in Excel format for post-processing. The statistical analysis of the data was implemented with the software IBM SPSS Statistics, version 27.

## 3. Results

### 3.1. Healthy Young Volunteers

One of the measures considered to be relevant was the mean applied force by the volunteers in the movement execution. The distribution of the acquired data shows that the male volunteers used the greater force, with an average of 124.0 N (with chair forward) and 136.5 N (with chair backward), compared with the results of the female volunteers, with an average of 91.0 N (with chair forward) and 93.5 N (with chair backwards), as shown in Table 3. FmaxRA Forward represents the maximal force of the right arm forward; FmaxLA Forward represents the maximal force of the left arm forward; FmaxRA Backward represents the maximal force of the right arm backward; FmaxLA Backward represents the maximal force of the left arm backward.

It can also be observed that there is no significant difference in the average forces made by each arm.

Additionally, *t*-tests of paired samples were carried out for both genders and arms. The results are presented in Table 4, where df is the abbreviation for degrees of freedom of the distribution t-student followed by the statistic test random variable. In all cases, the *p*-value was higher than 0.05, and therefore the equality of the arm strength was not rejected, except between the right arm strength forward and right arm strength backward. For this last case, since 0 belongs to the confidence interval, even though the *p*-value is 0.037, we did not consider that the forces were different, although it could be considered.

**Figure 4:**
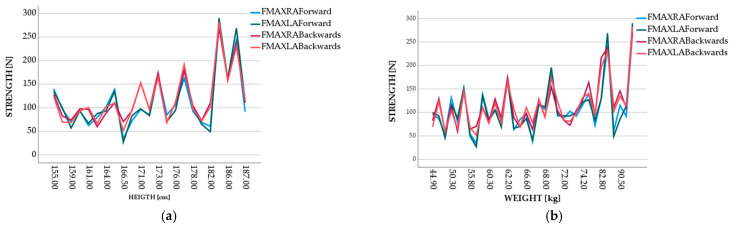
Visualization of graph: (**a**) height–strength; (**b**) weight–strength.

Figure 4 shows a relation between the weight of the volunteers and the average of the forces measured in tests performed, and a relation between the volunteers’ height and the maximum mean of the forces measured in the tests performed. In both situations, it can be observed that as the weight and the height increased, the peaks of the maximum of the applied forces also increased, which was expected.

A moderate correlation was observed between weight and applied forces (right arm: r = 0.403; left arm: r = 0.417) and height and applied forces (right arm: r = 0.409; left arm: r = 0.428).

### 3.2. Continuing Care Unit Volunteers

**Figure 5:**
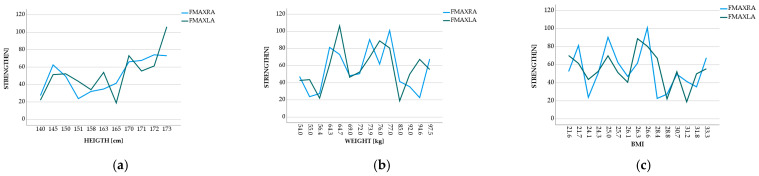
Visualization of graph: (**a**) height–strength; (**b**) weight–strength; (**c**) BMI–strength.

**Figure 6:**
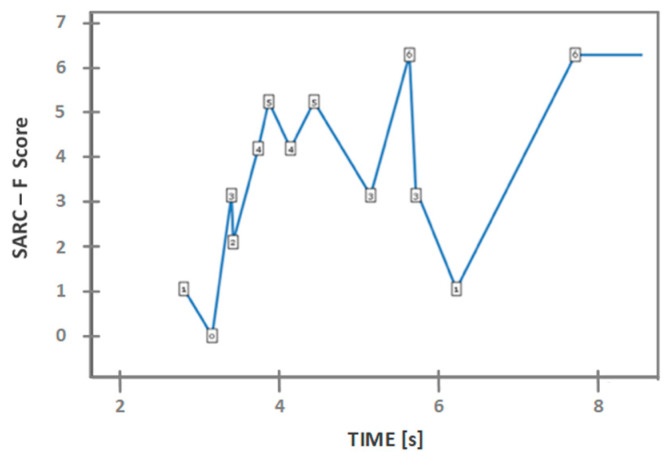
Visualization of graph SARC-F–time.

Figure 5 shows a relation between the maximum mean of the forces applied in the tests and the volunteers’ height, being able to identify an increasing trend. In other words, as the height increased, the maximum mean forces also increased. Figure 5 also shows a decreasing trend that can be seen between the mass of the volunteer and their body mass index—BMI concerning the strength, i.e., as the weight and the BMI increased, the applied force decreased. Figure 6 shows a relation between the SARC-F score and the time taken to perform the movement. As the SCARC-F increased, volunteers’ time to make the STS movement increased, although some outliers can be identified. Since sarcopenia is the loss of muscle mass and it is classified by score, i.e., the higher the score, the more predictive of having sarcopenia, it was expected that the volunteers with a superior score would take longer to make the STS movement.

A moderate correlation was observed between height and applied forces (right arm: r = 0.531; left arm: r = 0.559). A moderately high correlation between the SCAR-F score and time can also be seen (r = 0.546), as well as a negative correlation between the force applied by the left arm and the BMI (r = −0.333).

### 3.3. Healthy Young Volunteers vs. Continuing Care Unit Volunteers

Although the volunteers from the continuing care unit needed the device more to support themselves than the healthy young volunteers did, the healthy volunteers were asked to use the developed device as support, i.e., the volunteer felt that the device was support to stand up without requiring too much strength from the lower limbs. So, it would be expected that the maximum average strength of the healthy and young volunteers (both arms: F = 105 N) would be higher than that of the volunteer from the continuing care unit (right arm: F = 54 N; left arm: F = 56 N).

The relation between the maximum strength from the right arm and the maximum strength from the left arm was higher in healthy and young volunteers. This fact is due to the non-existence of differences between the forces measured for each arm.

## 4. Discussion and Conclusions

This work presents the development of a sensorized biomechanical device, which allows for quantifying and recording the applied force by the upper limbs during the “sit-to-stand” movement. The primary objective of the device was to understand some parameters associated with “sit-to-stand”, especially in individuals undergoing a motor and functional rehabilitation process.

This work involved the prototype’s conception, design and production and was tested in two different scenarios. One of the experimental tests was carried out by young and healthy volunteers. In the phase of transportation and installation of the device, it was noticeable how easy and quick it was to assemble it. The first result to be noticed by the device was its robustness in the domain of mechanical strength and stiffness. Additionally, the developed application and user interface have effectively acquired and recorded data associated with the movement. The device has shown its effectiveness both in observing force-time during the execution of the movement through the application interface developed and in recording data for post-processing.

The volunteers that carried out the other experimental tests were from a hospital continuing care unit. It should be considered that some of the volunteers needed to support themselves with technical aids to move. None of these volunteers had any limitations or restrictions on performing the STS movement with the developed device.

Regarding the sample acquired, we may conclude that:

- Male volunteers applied a more significant force than the female ones (with both arms and either forward or backward);
- The strength of both genders had superior values when applied with the chair backward;
- There were no significant differences in the average forces made by right or left arms;
- A moderate positive correlation exists between the strength applied and the weight and the strength and height of the individual.

It is worth mentioning that for the continuing care unit volunteers, there was a moderately high correlation between the SCAR-F score and the time taken to apply force, as well as a negative correlation between the force applied by the left arm and the BMI.

As expected, the maximum strength from the arms presents higher values in healthy and young volunteers. Additionally, it can be verified that the device identifies asymmetries in the applied forces in the STS movement between the upper limbs. From the application and the registered data, it can be observed that volunteers with motor problems in the lower limbs performed more strength in their arms to compensate, i.e., if a volunteer had a limitation associated with the left lower limb, they reveal more strength in the left upper limb.

Based on these results, the developed device can be a helpful tool for monitoring the evaluation of a patient with strengths and coordination limitations in the upper and lower limbs.

In addition, and as future work, a set of serious games are in implementation to allow the use of the device as a stimulus tool for the execution of the STS movement. We also wish to include a barometric platform that will allow us to measure the pressure and strength used in each lower limb.

The authors strongly believe that these developments in this device will allow better rehabilitation management in several health conditions, namely in strokes, Parkinson’s disease and geriatric populations with augmented risk of falls.

## Figures and Tables

**Table 1:** Data of the healthy volunteers.

Volunteer	Number	Age Mean—[Years]	BMI—Body Mass Index Mean—[kg/m^2^]	Weight Mean—[kg]	Height Mean—[cm]
Healthy	18 Male	22.31	23.36	67.93	169.78
	16 Female				

**Table 2:** Data of the volunteers from the hospital continuing care unit.

Volunteer	Number	Age Mean—[Years]	Gait Ability and Aid				Rehabilitation Process		
			None	Crutches	Walker	Wheelchair	Post-Stroke	Bone Fractures	Cerebral Hemorrhages
In Rehabilitation	10 Male 6 Female	71.06	7	2	3	4	8	5	3

Each one of the volunteers signed informed consent to participate in the tests. The Declaration of Helsinki followed all experimental procedures and data collection. Each volunteer was assigned an identification code, with no mention of personal data, so the confidentiality of the volunteers is guaranteed.

**Table 3:** Data descriptive statistics.

	Gender	Mean [N]	Std. Deviation	Std. Error Mean
FmaxRA Forward	M	123.4834	62.4835	14.7275
	F	92.2855	31.4182	7.8545
FmaxLA Forward	M	124.8073	71.3075	16.8073
	F	90.3186	31.2524	7.8131
FmaxRA Backward	M	136.9843	59.6426	14.0579
	F	93.6741	28.1382	7.0346
FmaxLA Backward	M	135.6057	60.6133	14.2867
	F	93.3576	25.6802	6.4201

**Table 4:** Paired sample *t*-tests.

		Paired Differences								
		Mean	Std. Deviation	Std. Error Mean	95% Confidence Interval of the Difference				Significance	
					Lower	Upper	t	df	One-Sided *p*	Two-Sided *p*
FmaxRA Forward vs. FmaxLA Backward		0.22	11.73	2.01	−3.87	4.32	0.112	33	0.456	0.912
FmaxRA Forward vs. FmaxLA Backward		0.88	11.25	1.93	−3.04	4.80	0.456	33	0.326	0.652
FmaxRA Forward vs. FmaxRA Backward		−7.80	24.70	4.24	−16.42	0.82	−1.841	33	0.037	0.075
FmaxLA Forward vs. FmaxLA Backward		−7.15	24.91	4.27	−15.84	1.55	−1.673	33	0.052	0.104

## Data Availability

Not applicable.
